# Eating from the wild: diversity of wild edible plants used by Tibetans in Shangri-la region, Yunnan, China

**DOI:** 10.1186/1746-4269-9-28

**Published:** 2013-04-19

**Authors:** Yan Ju, Jingxian Zhuo, Bo Liu, Chunlin Long

**Affiliations:** 1 College of Life and Environmental Sciences, Minzu University of China, Beijing 100081, PR China; 2 School of Agronomy and Biotechnology, Yunnan Agricultural University, Kunming 650201, PR China; 3 Kunming Institute of Botany, Chinese Academy of Sciences, Kunming 650201, PR China

**Keywords:** Wild edible plants, Traditional knowledge, Biodiversity, Ethnobotany, Shangri-la region

## Abstract

**Background:**

Locally harvested wild edible plants (WEPs) provide food as well as cash income for indigenous people and are of great importance in ensuring global food security. Some also play a significant role in maintaining the productivity and stability of traditional agro-ecosystems. Shangri-la region of Yunnan Province, SW China, is regarded as a biodiversity hotspot. People living there have accumulated traditional knowledge about plants. However, with economic development, WEPs are threatened and the associated traditional knowledge is in danger of being lost. Therefore, ethnobotanical surveys were conducted throughout this area to investigate and document the wild edible plants traditionally used by local Tibetan people.

**Methods:**

Twenty-nine villages were selected to carry out the field investigations. Information was collected using direct observation, semi-structured interviews, individual discussions, key informant interviews, focus group discussions, questionnaires and participatory rural appraisal (PRA).

**Results:**

Information about 168 wild edible plant species in 116 genera of 62 families was recorded and specimens were collected. Most species were edible greens (80 species) or fruits (78). These WEPs are sources for local people, especially those living in remote rural areas, to obtain mineral elements and vitamins. More than half of the species (70%) have multiple use(s) besides food value. Some are crop wild relatives that could be used for crop improvement. Several also have potential values for further commercial exploitation. However, the utilization of WEPs and related knowledge are eroding rapidly, especially in the areas with convenient transportation and booming tourism.

**Conclusion:**

Wild food plants species are abundant and diverse in Shangri-la region. They provide food and nutrients to local people and could also be a source of cash income. However, both WEPs and their associated indigenous knowledge are facing various threats. Thus, conservation and sustainable utilization of these plants in this area are of the utmost importance. Documentation of these species may provide basic information for conservation, possibly further exploitation, and will preserve local traditional knowledge.

## Background

Wild edible plants (WEPs) refer to species that are harvested or collected from their wild natural habitats and used as food for human consumption [1-3]. They provide staple food for indigenous people, serve as supplementary food for non-indigenous people and are one of the primary sources of cash income for poor communities [4-6]. WEPs play an important role in ensuring food security and improve the nutrition in the diets of many people in developing countries [1,5]. They are potential sources of species for domestication and provide valuable genetic traits for developing new crops through breeding and selection [7,8].

Although domesticated plants are the main source of food and income for people in rural areas, they are not able to meet the annual food requirements [9-11]. Thus, the collection and consumption of wild edible plants has been “a way of life” to supplement dietary requirements for many rural populations throughout the world [5,12]. However, due to social change and acculturation processes, indigenous knowledge (or traditional knowledge) about the use of wild edible species is declining and even vanishing with modernization and increasing contacts with western lifestyles [13]. Meanwhile, the loss of traditional knowledge has also been recognized as one of the major factors that have negative effects on the conservation of biological diversity [14]. Thus, it is becoming urgent to document and revitalize traditional knowledge of WEPs to preserve genetic and cultural diversity [12,15,16]. China is renowned for its wide use of wild harvested resources in the human diet, and many studies have focused on wild edible plants [17-28]. These ethnobotanical surveys not only play an important role in conserving traditional knowledge associated with WEPs, but also contribute to nutritional analysis of the most widely used species [1,13]. Nutritional analyses may provide significant information for the utilization of those species that have the best nutritional values, thus helping to maintain dietary diversity and improve local food security [1,2,15].

Diqing Tibetan Autonomous Prefecture of Yunnan Province, commonly known as the *Shangri-la region*, belongs to the world-famous area called Three Parallel Rivers (Nujiang River, Lancang River and Jinsha River). It is the core of the eastern Himalayas and is regarded as a biodiversity hotspot [29]. Because of its complex topography and high diversity of climates, abundant plant and animal species are distributed in this area [30,31]. Although Tibetans account for about 32.36% of the total population of the whole prefecture and have a relatively well-preserved and distinct cultural identity, there are also Lisu, Han, Naxi, Yi as well as Bai populations, among whom mutual cultural influences have existed for a long time [30,31]. Furthermore, the diet of local Tibetan people differs somewhat from that of Tibetans in Xizang Autonomous Region. People living in the Tibetan Plateau have a limited range of food choices. The staple traditional diet includes Tsampa (made from hull-less barley), yak meat, mutton, buttered tea, sweet tea, barley wine and yogurt [32]. They seldom eat vegetables or fruits. On the other hand, because plant resources in Diqing Prefecture are more plentiful, and local Tibetans are influenced by other nationalities, they not only cultivate various crops, but also collect wild edible plants as supplementary food. These WEPs provide various microelements, and are also an important feature of local agrobiodiversity in which Tibetans have traditionally lived. However, the ecology of Diqing Tibetan Autonomous Prefecture is very fragile, and agrobiodiversity is being rapidly lost due to many natural and human caused factors [33-35]. Many precious plant resources that may have potential for future sustainable development are vanishing before they have been discovered. The reduction of plant diversity also leads to the extinction of the associated indigenous knowledge [36]. Thus, documentation and evaluation of edible plants and relevant local knowledge is urgently needed. This work may guide proper conservation and sustainable utilization of those wild food plants and related indigenous knowledge.

Although there are several ethnobotanical studies concerning wild food plants used by ethnic minorities, such as Mongolians [18,19], Miao in Hunan Province [21] and various ethnic groups in Yunnan Province [5,17,26-28], to our knowledge, information on WEPs of the Shangri-la region used by Tibetans has not previously been documented. In order to fill this gap, ethnobotanical surveys were conducted throughout the prefecture. Scientific and local names, plant parts used, modes of preparation, seasonality patterns in collection and use, and commercialization possibilities of the WEPs are presented in this paper.

## Methods

### Study area

**Figure 1:**
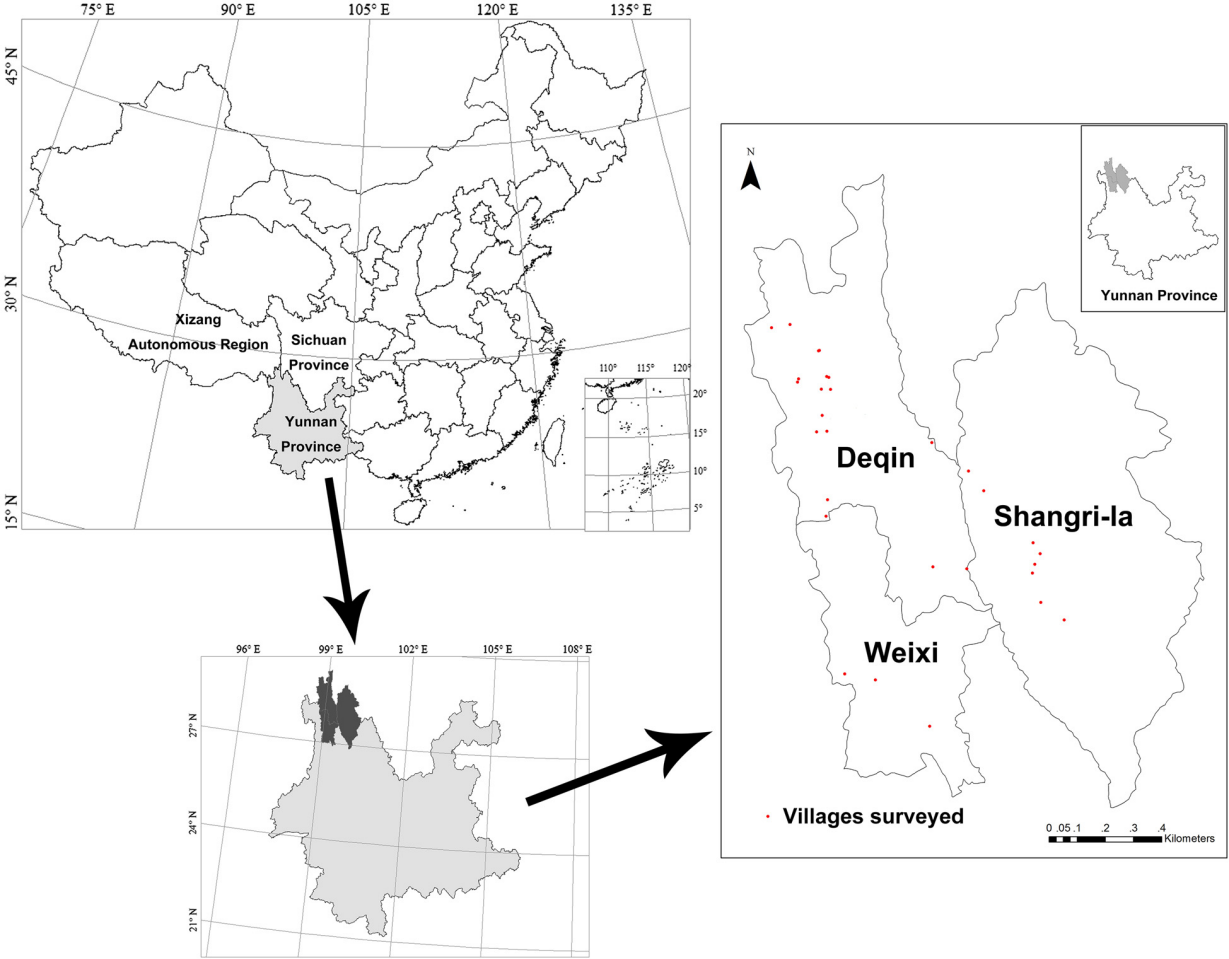
Location of the area covered in an investigation into the wild edible plants used by Tibetans in the Shangri-la region, Yunnan, China.

The study was carried out in Diqing Tibetan Autonomous Prefecture, northwest Yunnan, situated in the south of the Qinghai-Tibet Plateau of the eastern Himalayas, at the junction of Yunnan, Tibet and Sichuan Provinces (between 98°35’-100°19’ E and 26°52’-29°16’ N) (Figure 1). Three counties, Shangri-la, Deqin and Weixi are administered by the prefecture, with a total area of 23,870 square kilometers and a population of about 400,000. The terrain is higher in the north and lower in the south. The lowest altitude, 1,480 m is at the junction of the Biyu and Lancang Rivers in Weixi County, and the highest altitude, 6,740 m is Kawagebo Peak of the Meili Snow Mountains. The climate of Diqing is divided into five zones: 1) northern subtropical and warm temperate (below 2500 m); 2) temperate (2500–3000 m); 3) cold temperate (3000–4000 m); 4) frigid (4000–5000 m); and 5) glacier (above 5000 m). Abundant plant resources are distributed in this area because of its unique geographical location and climate diversity [31].

### Field survey and data collection

Prior to our field work, relevant literature was consulted to obtain information on the topography, climate, and local culture of Diqing Prefecture, this was helpful in choosing the specific study sites [31]. Field studies were carried out during three visits in March, July and August, 2012. After considering the terrain and climate condition, 29 villages belonging to three counties (8 in Shangri-la, 3 in Weixi and 18 in Deqin) and located in high mountains as well as lower river valleys were randomly selected to carry out ethnobotanical investigation (Table 1). Two-hundred and eighty-two randomly selected households (eight to ten people per village) were surveyed. Ethnobotanical data were collected through different interview methods (participatory rural appraisal (PRA), direct observation, semi-structured interviews, key informant interviews, individual discussions, focus group discussions and questionnaires) [37-40].

**Table 1:** Villages surveyed in investigations of wild edible plants used by Tibetans in Shangri-la region, Yunnan Province, China

**No.**	**Name of village**	**Latitude (north)**	**Longitude (east)**	**Altitude(m)**
1	Laza Village, Shangri-la County	27°45’39.6”	99°40’22.8”	3320
2	Jiefang Village, Shangri-la County	27°51’46.8”	99°41’56.4”	3280
3	Nishi Village, Shangri-la County	27°47’21.8”	99°40’59.5”	3290
4	Kaisong Village, Shangri-la County	27°53’49.6”	99°38’21.5”	3270
5	Dala Village, Shangri-la County	27°31’12”	99°57’36”	3370
6	Xiaozhongdian Village, Shangri-la County	27°34’12”	99°47’59”	3260
7	Xingfu Village, Shangri-la County	28°8’27.6”	99°25’58.8”	2230
8	Nixi Village, Shangri-la County	28°4’1.2”	99°29’34.8”	3170
9	Laohao Village, Weixi County	27°10’54.1”	99°17’20.4”	2260
10	Gongyuan Village, Weixi County	27°21’50.35”	99°5’11.45”	1690
11	Biluo Village, Weixi County	27°25’22.8”	99°1’58.8”	2630
12	Feilaisi Village, Deqin County	28°26’31.2”	98°52’44.4”	3390
13	Wunongding Village, Deqin County	28°26’56.4”	98°54’46.8’	3530
14	Mingyong Village, Deqin County	28°28’8.4”	98°47’42”	2270
15	Adunzi Village, Deqin County	28°29’13.6”	98°54’38.9”	3290
16	Gusong Village, Deqin County	28°29’37.7”	98°54’10.1”	3590
17	Adong Village, Deqin County	28°45’46.8”	98°39’14.4”	2690
18	Hongpo Village, Deqin County	28°17’2.4”	98°54’18”	2810
19	Guonian Village, Deqin County	28°17’16.71”	98°51’49.61”	2130
20	Jiulongding Village, Deqin County	28°20’42”	98°53’13.2”	2570
21	Sinong Village, Deqin County	28°29’9.18’	98°47’33.42’	2320
22	Badong Village, Deqin County	27°57’39.6”	98°54’0”	2240
23	Cizhong Village, Deqin County	28°01’16.44’	98°54’16.14’	1970
24	Gongka Village, Deqin County	28°35’27.6”	98°52’12”	3080
25	Jiunong Village, Deqin County	28°43’28.77”	98°41’4.76”	3160
26	Luwa Village, Deqin County	28°40’30”	98°41’38.4”	2290
27	Xiaruo Village, Deqin County	27°48’3.77”	99°18’5.11”	2040
28	Tuoding Village, Deqin County	27°46’10.9’	99°25’37.2’	1940
29	Benzilan Village, Deqin County	28°14’36.83”	99°18’7.43”	2150

During our survey, the local Tibetan pronunciations, parts used, collection period and preparation methods plants were recorded. Because local Tibetan pronunciations differ from the formal Tibetan pronunciation of Xizang Autonomous Region, and the names of some species were even pronounced the same as in Mandarin Chinese, we recorded the names phonetically exactly as they were spoken to us. Most Tibetans in Diqing Prefecture, especially the official workers, students and traders can speak basic Mandarin, therefore our interviews were in Mandarin and did not use interpreters.

Specimens were examined and identified by the authors and other taxonomists and will be deposited in the Herbarium of the Minzu University of China (Beijing).

## Results and discussion

### Wild food plant diversity and frequently utilized species

The study area is floristically rich and has a large number of useful WEP species. The 168 species documented include angiosperms (153 spp.), gymnosperms (4), pteridophytes (4), algae (2) and lichens (5) (Table 2), of which 41.1% are endemic to China and 11.9% endemic to northwestern Yunnan Province. Details of utilization are given in Table 3 (plants mentioned only by one informant are not documented in this list). The average number of species mentioned per informant is around ca. 8 species. Plants belonging to 62 families and 116 genera are distributed into different life forms, with herbs (43.5%) and shrubs (26.8%) having the most species, similar to a survey conducted in Yunnan Province [17] and another in Hunan Province [21]. The majority of food plants belong to the Rosaceae (34 species), Liliaceae (9), Brassicaceae (9), Araliaceae (6) and Berberidaceae (6). The genera represented by the highest number of species are *Rubus* (8 species), followed by *Maianthemum* (6), *Berberis* (4), *Cornus* (4), *Lindera* (4) and *Pyrus* (4).

**Table 2:** Taxonomic distribution of wild edible plants used by Tibetans in Shangri-la region, Yunnan Province, China

**Plant group**	**Number of species**	**Number of genera**	**Number of families**
Angiosperm	153	101	47
Gymnosperm	4	4	4
Pteridophyte	4	4	4
Algae	2	2	2
Lichen	5	5	5
Total	168	116	62

**Table 3:** Wild edible plants used by the Tibetans in Shangri-la region, Yunnan Province, China

**Latin name**	**Local name**	**Family name**	**Distribution**	**Parts used**	**Local use (edible only)**	**Collection period**	**Additional local use(s)**	**Frequency**
*Actinidia arguta* (Siebold et Zucc.) Planch. ex Miq.	*Zhemenkoubu*	Actinidiaceae	Shangri-la, Weixi and Deqin	Fruits	ripe fruits eaten fresh.	Aug-Sept	Whole plants used as hedge plants.	***
*Actinidia pilosula* (Finet et Gagnep.) Stapf ex Hand.-Mazz.	*Zhemenkoubu*	Actinidiaceae	Shangri-la, Weixi and Deqin	Fruits	ripe fruits eaten fresh.	Aug-Sept	Whole plants used as hedge plants.	**
*Actinidia venosa* Rehder	*Zhemenkoubu*	Actinidiaceae	Shangri-la, Weixi and Deqin	Fruits	ripe fruits eaten fresh.	Aug-Sept	Whole plants used as hedge plants.	**
*Adenophora khasiana* (Hook. f. et Thomson) Collett et Hemsl.	*Zheibamiedu*	Campanulaceae	Weixi and Deqin	Roots	stewed with meat and eaten as tonic.	Jul-Sept	Flowers and stems used for *weisang*. Aerial parts used as fodder. Roots used to treat cough and clearing heat.	***
*Alectoria sulcata* Nyl.	*Shuhua*	Usneaceae	Shangri-la, Weixi and Deqin	Whole plant	stir-fried	Jul-Sep		*
*Allium hookeri* Thwaites var*. muliense* Airy-Shaw	*Rijiucai*	Liliaceae	Shangri-la, Weixi and Deqin	Aerial parts	stir-fried or added to soups	May-Aug		***
*Allium ovalifolium* Hand.-Mazz.	*Rijiucai*	Liliaceae	Shangri-la, Weixi and Deqin	Aerial parts	stir-fried or added to soups	May-Aug		*****
*Allium trifurcatum* (F. T. Wang et T. Tang) J. M. Xu	*Rijiucai*	Liliaceae	Shangri-la, Weixi and Deqin	Aerial parts	stir-fried or added to soups	May-Aug		****
*Amaranthus caudatus* L.	*Yani*	Amaranthaceae	Shangri-la, Weixi and Deqin	Young stems and leaves	stir-fried or added to soups	Jun-Jul	Aerial parts used as fodder.	***
*Amaranthus hypochondriacus* L.	*Yani*	Amaranthaceae	Shangri-la, Weixi and Deqin	Young stems and leaves	stir-fried or added to soups	Jun-Jul	Aerial parts used as fodder.	***
*Amygdalus mira* (Koehne) Ricker	*Yemaotao; Kamu*	Rosaceae	Shangri-la, Weixi and Deqin	Fruits	eaten fresh.	Jul-Aug	Seeds used to relieve a cough and cure injuries.	***
*Anemone rivularis* Buch.-Ham. ex DC.	*Huzhangcao*	Ranunculaceae	Weixi	Roots	stewed with meat and eaten as tonic	Jun-Sept	Roots used to treat bronchitis. Whole plant used as ornamental.	*
*Aralia caesia* Hand.-Mazz.	*Shutoucai*	Araliaceae	Shangri-la	Young leaves and leaf buds	stir-fried or eaten fresh	Apr-May		****
*Aralia chinensis* L.	*Gege*	Araliaceae	Shangri-la, Weixi and Deqin	Young leaves and leaf buds	stir-fried or eaten fresh	Apr-May	Bark used for *weisang*	*****
*Arctium lappa* L.	*Baomujicigen*	Asteraceae	Shangri-la, Weixi and Deqin	Roots	stewed with meat and eaten as tonic.	Jun-Aug	Fruits, leaves and roots used to relieve fever, and treat measles, dysentery and gastropathy.	***
*Arisaema erubescens* (Wall.) Schott	*Reduo*	Araceae	Shangri-la, Weixi and Deqin	Young leaves	stir-fried	Jun-Jul	Tubers used to relieve cough and treat hemoptysis and pneumonia.	**
*Aristolochia delavayi* Franch.	*Ricaoko*	Aristolochiaceae	Shangri-la	Whole plants	stir-fried and used as spice	Aug-Sept	Whole plants used as stomachic tonic.	***
*Armeniaca mume* Siebold	*Kangjue*	Rosaceae	Shangri-la, Weixi and Deqin	Fruits	eaten fresh.	Aug	Used as rootstock for *Armeniaca vulgaris.*	*
*Arundinaria faberi* Rendle	*Sunzi*	Poaceae	Shangri-la, Weixi and Deqin	New shoots	boiled or stir-fried	Jul-Aug	Aerial parts used as fodder and to make bamboo wares.	*****
*Berberis amoena* Dunn	*Qiesi*	Berberidaceae	Shangri-la, Weixi and Deqin	Young stems, leaves and fruits	eaten fresh	May-Sep	Whole plants used as fence and hedge plants.	***
*Berberis jamesiana* Forrest et W. W. Sm.	*Qiesi*	Berberidaceae	Shangri-la, Weixi and Deqin	Young stems, leaves and fruits	eaten fresh	May-Sep	Whole plants used as fence and hedge plants.	***
*Berberis pruinosa* Franch.	*Qiesi*	Berberidaceae	Shangri-la, Weixi and Deqin	Young stems, leaves and fruits	eaten fresh	May-Sep	Whole plants used as fence and hedge plants.	***
*Berberis weisiensis* C. Y. Wu ex S. Y. Bao	*Qiesi*	Berberidaceae	Shangri-la, Weixi and Deqin	Young stems, leaves and fruits	eaten fresh	May-Sep	Whole plants used as fence and hedge plants.	**
*Berchemia hirtella* Tsai et K. M. Feng	*Zhila*	Rhamnaceae	Deqin	Fruits	eaten fresh	Aug-Sep		**
*Berchemia hirtella* Tsai et K. M. Feng	*Zhila*	Rhamnaceae	Deqin	Young leaves	used for making tea	Apr-Jun		**
*Berchemia sinica* C. K. Schneid.	*Zhila*	Rhamnaceae	Deqin	Fruits	eaten fresh	Aug-Sep		**
*Berchemia sinica* C. K. Schneid.	*Zhila*	Rhamnaceae	Deqin	Young leaves	used for making tea	Apr-Jun		**
*Boehmeria penduliflora* Wedd. ex Long	*Sejia*	Urticaceae	Deqin	Young stems and leaves	boiled or stir-fried	Jun-Jul		*
*Boehmeria tricuspis* (Hance) Makino	*Sejia*	Urticaceae	Deqin	Young stems and leaves	stir-fried	Jun-Jul		*
*Broussonetia papyrifera* (L.) L'Hér. ex Vent.		Moraceae	Shangri-la, Weixi and Deqin	Fruits	eaten fresh	Sep-Oct	Leaves used as fodder. Bark used for papermaking.	*
*Capsella bursa-pastoris* (L.) Medik.	*Zijisuona*	Brassicaceae	Shangri-la, Weixi and Deqin	Aerial part	stir-fried	May-Jun	Aerial parts used as fodder.	**
*Cardamine yunnanensis* Franch.	*Lijisuona*	Brassicaceae	Shangri-la, Weixi and Deqin	Aerial part	stir-fried	May-Jun	Aerial parts used as fodder.	**
*Cephalotaxus fortunei* Hook. var. *alpina* H. L. Li	*Miyou*	Cephalotaxaceae	Weixi, Deqin	Seeds	eaten fresh or stir-fried	Sep-Oct	Plants used as fuel-wood. Seeds used to expel parasite.	*
*Cerasus conadenia* (Koehne) T. T. Yu et C. L. Li	*Xumumiedu*	Rosaceae	Shangri-la, Weixi and Deqin	Fruits	eaten fresh	Jul-Aug	Flowers and leaves used for *weisang*	***
*Cerasus tomentosa* (Thunb.) Wall.	*Nuosi*	Rosaceae	Shangri-la, Weixi and Deqin	Fruits	eaten fresh	Jul-Sept		**
*Chaenomeles speciosa* (Sweet) Nakai	*Suomugua*	Rosaceae	Shangri-la, Weixi and Deqin	Fruits	stewed with meat as spice and used to prepare local wine	Sept-Oct		***
*Chenopodium album* L.	*Hui*	Chenopodiaceae	Shangri-la, Weixi and Deqin	Young stems and leaves	stir-fried	Jun-Jul	Aerial parts used as fodder.	***
*Cinnamomum glanduliferum* (Wall.) Meisner	*Xiangzhangzi*	Lauraceae	Shangri-la, Weixi and Deqin	Fruits	stir-fried and used as spices	Aug-Sept	Fruits used to treat stomachache.	*
*Cirsium japonicum* (Thunb.) Fisch. ex DC.	*Baimaci*	Asteraceae	Shangri-la, Weixi and Deqin	Roots	stewed with meat and eaten as tonic	Jun-Aug	Young stems and leaves used as fodder.	***
*Codonopsis pilosula* (Franch.) Nannf. var. *handeliana* (Nannf.) L. T. Shen	*Dangshen*	Campanulaceae	Deqin and Weixi	Roots	stewed with meat and eaten as tonic	Jul-Sept	Aerial parts used as fodder. Roots used to invigorate the spleen.	***
*Coriaria nepalensis* Wall.	*Masen*	Coriariaceae	Weixi	Fruits	eaten fresh	May-Jun		*
*Cornus capitata* Wall.	*Jisuo; Jisuziguo*	Cornaceae	Shangri-la, Weixi and Deqin	Fruits	eaten fresh	Aug-Sept	Fruits, stems and leaves used as veterinary medicine.	***
*Cornus macrophylla* Wall.	Dengtaishu	Cornaceae	Shangri-la, Weixi and Deqin	Seeds	used for making vegetable oil.	Aug-Sept	Plants used as fuel-wood.	**
*Cornus schindleri* Wangerin	Saisaizi	Cornaceae	Shangri-la, Weixi and Deqin	seeds	used for making vegetable oil	Aug-Sept	Plants used as fuel-wood.	**
*Cornus ulotricha* C. K. Schneid. et Wangerin	Dengtaishu	Cornaceae	Shangri-la, Weixi and Deqin	seeds	used for making vegetable oil	Aug-Sept	Plants used as fuel-wood.	*
*Corylus chinensis* Franch.	*Jilizi*	Betulaceae	Shangri-la, Weixi and Deqin	Fruits	used for making pastries	Sept-Oct	Wood used for construction or furniture.	**
*Corylus yunnanensis* (Franch.) Camus	*Shanbaiguo*	Betulaceae	Shangri-la, Weixi and Deqin	Fruits	used for making pastries	Sept-Oct	Woods used for construction or furniture.	*
*Cotinus coggygria* Scop. var. *glaucophylla* C. Y. Wu	*Jiade*	Anacardiaceae	Shangri-la	Young leaves	boiled or stir-fried	May-Jun	Whole plants used as ornamental.	*
*Crataegus chungtienensis* W. W. Sm.	*Lubu*	Rosaceae	Weixi and Shangri-la	Fruits	eaten fresh	Sept	Whole plants used as fence and hedge plants.	***
*Crataegus oresbia* W. W. Sm.	*Lubu*	Rosaceae	Weixi and Shangri-la	Fruits	eaten fresh	Aug-Sept	Whole plants used as fence and hedge plants.	***
*Cynanchum forrestii* Schltr.	*Babeda*	Asclepiadaceae	Deqin and Weixi	Fruits	eaten fresh	Aug-Oct	Roots stewed with meat and eaten to treat rheumatism.	*
*Davidia involucrata* Baill. var. *vilmoriniana* (Dode) Wangerin	*Labizi*	Nyssaceae	Weixi	Fruits	eaten fresh	Sept-Oct	Whole plant used as ornamental.	*
*Debregeasia orientalis* C. J. Chen	*Jiaojia*	Urticaceae	Shangri-la, Weixi and Deqin	Fruits	eaten fresh and used to make local wine	Jun-Aug	Roots used to treat rheumatoid arthritis and broken bones.	*
*Decaisnea insignis* (Griff.) Hook. f. et Thomson	*Xianli*	Lardizabalaceae	Shangri-la, Weixi and Deqin	Fruits	eaten fresh and used to make local wine	Jul-Aug	Roots and fruits used to clearing heat.	***
*Dioscorea melanophyma* Prain et Burkill	*Huangshayue*	Dioscoreaceae	Weixi	Tubers	boiled or stir-fried	Jun-Jul	Aerial parts used as fodder.	**
*Diospyros lotus* L.	*Tazhi*	Ebenaceae	Shangri-la, Weixi and Deqin	Fruits	eaten fresh	Sept-Oct		***
*Duchesnea indica* (Andrews) Focke	*Dihongpao*	Rosaceae	Shangri-la, Weixi and Deqin	Fruits	eaten fresh	Jun-Jul		*
*Elaeagnus multiflora* Thunb.	*Cibie*	Elaeagnaceae	Shangri-la, Weixi and Deqin	Fruits	eaten fresh	Jun-Jul		**
*Elaeagnus umbellata* Thunb.	*Yangnaiguo*	Elaeagnaceae	Shangri-la, Weixi and Deqin	Fruits	eaten fresh	Jul-Aug		**
*Eriobotrya salwinensis* Hand.-Mazz.		Rosaceae	Weixi and Deqin	Fruits	eaten fresh	Jun-Aug	Plants used as fuel-wood.	*
*Eutrema deltoideum* (Hook. f. et Thomson) O. E. Schulz	*Limo*	Brassicaceae	Shangri-la, Weixi and Deqin	Young stems and leaves	stir-fried	May-Jun	Aerial parts used as fodder.	**
*Eutrema heterophyllum* (W. W. Sm.) H. Hara	*Limo*	Brassicaceae	Shangri-la, Weixi and Deqin	Young stems and leaves	stir-fried	May-Jun	Aerial parts used as fodder.	**
*Eutrema himalaicum* Hook. f. et Thomson	*Limo*	Brassicaceae	Shangri-la, Weixi and Deqin	Young stems and leaves	stir-fried	May-Jun	Aerial parts used as fodder.	**
*Fagopyrum dibotrys* (D. Don) H. Hara	*Wanao*	Polygonaceae	Deqin	Young stems and leaves	stir-fried	Jun-Aug	Aerial parts used as fodder.	*
*Fargesia melanostachys* (Hand.-Mazz.) T. P. Yi	*Sunzi*	Poaceae	Shangri-la, Weixi and Deqin	New shoots	boiled or stir-fried	May-Aug	Aerial parts used as fodder and to make bamboo wares.	*****
*Ficus pumila* L.	*Dongshili*	Moraceae	Shangri-la, Weixi and Deqin	Fruits	used for making bean jelly	Jul-Aug	Leaves used as fodder.	*
*Ficus sarmentosa* Buch.-Ham. ex Sm.	*dongshili*	Moraceae	Shangri-la, Weixi and Deqin	Fruits	used for making bean jelly	Jul-Aug	Leaves used as fodder.	*
*Foeniculum vulgare* Mill.	*Asi*	Apiaceae	Shangri-la, Weixi and Deqin	Young stems and leaves	eaten fresh or stir-fried	May-Jul		**
*Fragaria moupinensis* (Franch.) Cardot	*Gasuo*	Rosaceae	Shangri-la, Weixi and Deqin	Fruits	eaten fresh.	Jun-Jul	Whole plants used as fodder.	**
*Galinsoga parviflora* Cav.	*Nawabijia*	Asteraceae	Deqin and Weixi	Young stems and leaves	boiled or stir-fried	Jun-Aug	Whole plants used as fodder.	*
*Ginkgo biloba* L.	*Baiguo*	Ginkgoaceae	Deqin, Weixi	Seeds	eaten fresh or stir-fried	Sep-Oct	Seeds used to treat asthma.	*
*Gnaphalium affine* D. Don	*Qingmincai*	Asteraceae	Weixi	Young leaves	grounded with sticky rice to make rice cake.	Apr-May	Leaves used to treat cuts and gun shot wounds.	*
*Herminium lanceum* (Thunb. ex Sw.) Vuijk	*Lianxiongde*	Orchidaceae	Shangri-la	Whole plant	stewed with meat and eaten as tonic.	Aug-Sep	Whole plant used as fodder.	*
*Hippophae rhamnoides* L. subsp. *yunnanensis* Rousi	*Xiju*	Elaeagnaceae	Deqin. Shangri-la	Fruits	eaten fresh or used to make beverage and wine.	Aug-Oct	Fruits used to treat cough and invigorate the circulation of blood.	*****
*Houttuynia cordata* Thunb.	*Zhergen*	Saururaceae	Weixi, Shangri-la	Leaves and roots	eaten fresh or stir-fried	Jun-Jul		***
*Juglans regia* L.	*Daiga*	Juglandaceae	Shangri-la, Weixi and Deqin	Seeds	eaten fresh or stir-fried, and used for making vegetable oil.	Aug-Sept	Plants used as fuel-wood.	***
*Kalopanax septemlobus* (Thunb.) Koidz.	*Cilaobao*	Araliaceae	Shangri-la, Weixi and Deqin	Young stems and leaves	eaten fresh or stir-fried	May-Jun		**
*Lethariella cladonioides* (Nyl.) Krog	*Gangge*	Parmeliaceae	Deqin	Whole plant	used for making tea, wine and beverage	Aug-Oct	Used to tranquilize mind and clearing heat.	*
*Leycesteria formosa* Wall.	*Sezha*	Caprifoliaceae	Deqin	Fruits	eaten fresh.	Aug-Oct		*
*Ligusticum daucoides* (Franch.) Franch.	*Riqincai*	Apiaceae	Shangri-la	Whole plants	stir-fried or added to soups	Apr-May	Aerial parts used as fodder.	****
*Lindera kariensis* W. W. Sm.	*Rihujiao*	Lauraceae	Weixi, Deqin	Fruits	used as spices	Jul-Sept		**
*Lindera nacusua* (D. Don) Merr.	*Rihujiao*	Lauraceae	Weixi	Fruits	used as spices	Jul-Sept		**
*Lindera obtusiloba* Blume var. *heterophylla* (Meisn.) H. P. Tsui	*Rihujiao*	Lauraceae	Weixi	Fruits	used as spices	Jul-Sept		*
*Lindera reflexa* Hemsl.	*Rihujiao*	Lauraceae	Weixi	Fruits	used as spices	Jul-Sept		*
*Lobaria* sp.	*Qingwapi*	Stictaceae	Shangri-la and Weixi	Aerial part	eaten fresh	Jul-Sept	Whole plant used to treat dyspepsia.	*
*Lycopus lucidus* Turcz. ex Benth.	*Ganluo*	Lamiaceae	Shangri-la	Young stems and leaves	eaten fresh or stir-fried or used for making pickle	Jul-Aug		**
*Mahonia duclouxiana* Gagnep.	*Jisa*	Berberidaceae	Deqin	Fruits	eaten fresh.	Aug-Sep	Whole plants used as hedge plants.	*
*Maianthemum atropurpureum* (Franch.) LaFrankie	*Zhuyecai;* *Nibai*	Liliaceae	Shangri-la, Weixi and Deqin	Young shoots and leaves	stir-fried or added to soups	May-Jun	Aerial parts used as fodder.	*****
*Maianthemum forrestii* (W. W. Smith) LaFrankie	*Zhuyecai;* *Nibai*	Liliaceae	Shangri-la and Weixi	Young shoots and leaves	stir-fried or added to soups	May-Jun	Aerial parts used as fodder.	****
*Maianthemum henryi* (Baker) LaFrankie	*Zhuyecai;* *Nibai*	Liliaceae	Shangri-la, Weixi and Deqin	Young shoots and leaves	stir-fried or added to soups	May-Jun	Aerial parts used as fodder.	****
*Maianthemum oleraceum* (Baker) LaFrankie	*Zhuyecai;* *Nibai*	Liliaceae	Weixi, Shangri-la	Young shoots and leaves	stir-fried or added to soups	May-Jun	Aerial parts used as fodder.	***
*Maianthemum purpureum* (Wallich) LaFrankie	*Zhuyecai;* *Nibai*	Liliaceae	Shangri-la, Weixi and Deqin	Young shoots and leaves	stir-fried or added to soups	May-Jun	Aerial parts used as fodder.	*****
*Maianthemum tatsienense* (Franct.) LaFrankie	*Zhuyecai;* *Nibai*	Liliaceae	Shangri-la	Young shoots and leaves	stir-fried or added to soups	May-Jun	Aerial parts used as fodder.	****
*Malus rockii* Rehder	*Tangli*	Rosaceae	Shangri-la, Weixi and Deqin	Fruits	eaten fresh	Sept	Plants used as fuel-wood, and rootstock for *Malus pumila.* Whole plants used as fence.	***
*Malus spectabilis* (Ait.) Borkh.	*Haitangguo*	Rosaceae	Shangri-la, Weixi and Deqin	Fruits	eaten fresh	Aug-Sept	Fruits decoction used to treat dark urine.	**
*Malva verticillata* L.	*Jiangba*	Malvaceae	Deqin	Young stems and leaves	stir-fried	Jun-Aug	Leaves, stems and seeds used as fodder. Whole plant used as ornamental.	*
*Matteuccia struthiopteris* (L.) Tadaro	*Huangguaxiang*	Onocleaceae	Shangri-la, Weixi and Deqin	Immature fronds	eaten fresh or stir-fried	May-Jun		****
*Medicago lupulina* L.	*Mocuo*	Fabaceae	Deqin, Shangri-la	Young stems and leaves	eaten fresh or stir-fried	Jun-Jul	Leaves, stems, flowers and seeds used as fodder.	*
*Megacarpaea delavayi* Franch.	*Yuose*	Brassicaceae	Shangri-la, Weixi and Deqin	Young stems and leaves	stir-fried	May-Jun	Aerial parts used as fodder.	**
*Megacarpaea polyandra* Benth. ex Madden	*Yuose*	Brassicaceae	Shangri-la, Weixi and Deqin	Young stems and leaves	stir-fried	May-Jun	Aerial parts used as fodder.	**
*Mentha canadensis* L.	*Qiubi*	Lamiaceae	Shangri-la, Weixi and Deqin	Young leaves	eaten fresh or stir-fried	Jun-Aug		**
*Nostoc sphaerioides* Kützing	*Shuimuer*	Nostocaceae	Shangri-la	Whole plant	eaten fresh or added to soups	Jun-Jul	Whole plant used to treat burns and scalds.	*
*Metapanax delavayi* (Franch.) J. Wen et Frodin		Araliaceae	Deqin, Weixi	Young leaves	used for making tea	Apr-May	Whole plants used as hedge plants.	*
*Ophioglossum reticulatum* L.	*Yimuyidun*	Ophioglossaceae	Shangri-la	Immature fronds	stir-fried or added to soups	Jul-Aug	Whole plants used to treat impotence and lumbago.	*
*Opuntia monacantha* (Willd.) Haw.	*Xianrenguo*	Cactaceae	Shangri-la, Weixi and Deqin	Fruits	eaten fresh	Aug-Sep	Tubers and fruits used as fodder. Whole plants used as fence and hedge plants.	***
*Oreorchi*s *indica* (Lindl.) Hook. f.	*Xiabaji*	Orchidaceae	Shangri-la, Weixi and Deqin	Pseudobulbs	boiled or stir-fried	Jun-Aug	Whole plants used as fodder. Pseudobulbs used to stop bleeding and detumescence.	*
*Osmunda japonica* Thunb.	*Shuijuecai*	Osmundaceae	Weixi	Immature fronds	stir-fried	May-Jun		***
*Osteomeles schwerinae* C. K. Schneid.	*Sele*	Rosaceae	Shangri-la, Weixi and Deqin	Fruits	eaten fresh	Aug-Sept	Leaves and fruits used as fodder.	**
*Panax japonicus* (T. Nees) C. A. Meyer var. *major* (Burkill) C. Y. Wu et K. M. Feng	*Gedeqi*	Araliaceae	Shangri-la	Young stems and leaves	eaten fresh or stir-fried	May-Jun	Whole plants used as fodder. Roots used to stop bleeding.	***
*Panax japonicus* (T. Nees) C. A. Meyer var. *major* (Burkill) C. Y. Wu et K. M. Feng	*Gedeqi*	Araliaceae	Shangri-la	Rhizomes	stewed with meat and eaten as tonic.	Jul-Aug	Whole plants used as fodder. Rhizomes used to stop bleeding.	***
*Pentapanax henryi* Harms		Araliaceae	Shangri-la, Weixi and Deqin	Young stems and leaves	eaten fresh or stir-fried	Apr-May		**
*Photinia glomerata* Rehder et E. H. Wilson	*Chongsi*	Rosaceae	Deqin	Fruits	eaten fresh	Sept		*
*Phyllanthus emblica* L.	*Ganlan*	Euphorbiaceae	Shangri-la	Fruits	eaten fresh	Jul-Sept	Barks used to extract tannin.	***
*Phytolacca acinosa* Roxb.	*Tuoqiong*	Phytolaccaceae	Deqin	Young stems and leaves	eaten fresh or stir-fried	Jul-Aug	Roots used to promote diuresis.	*
*Pinellia pedatisecta* Schott	*Luoa*	Araceae	Deqin	Young leaves	stir-fried	Jun-Jul	Corms used to treat vomit and http://reducehttp://phlegm.	*
*Pinus armandii* Franch.	*Seitu; Songzi*	Pinaceae	Shangri-la, Weixi and Deqin	Seeds	eaten fresh or stir-fried	Sept-Oct	Leaves and stems used for *weisang.* Needles used as fodder. Plants used as fuel-wood.	**
*Pistacia weinmanniifolia* J. Poiss. ex Franch.	*Li*	Anacardiaceae	Deqin	Fruits	eaten fresh	Aug-Sept	Leaves and stems used for *weisang*. Leaves and fruits used as fodder.	****
*Plantago asiatica* L.	*Hamaye*	Plantaginaceae	Shangri-la, Weixi and Deqin	Whole plants	boiled or stir-fried	Jun-Aug	Leaves, stems, flowers and seeds used as fodder.	***
*Plantago major* L.	*Hamaye*	Plantaginaceae	Shangri-la, Weixi and Deqin	Whole plants	boiled or stir-fried	Jun-Aug	Leaves, stems, flowers and seeds used as fodder.	***
*Potentilla anserina* L.	*Chuomo*	Rosaceae	Shangri-la, Weixi and Deqin	Roots	eaten fresh or boiled	Jun-Sept	Leaves, stems and fruits used as fodder. Roots used to control leukorrhea flow.	***
*Potentilla coriandrifolia* D. Don var. *dumosa* Franch.	*Zumuyasha*	Rosaceae	Shangri-la, Weixi and Deqin	Roots	eaten after boiling	Jun-Sept	Whole plants used as fodder.	*
*Potentilla leuconota* D. Don	*Pagu*	Rosaceae	Shangri-la, Weixi and Deqin	Roots	eaten after boiling	Jun-Sept	Whole plants used as fodder.	*
*Prasiola subareolata* Skuja.	*Shihuacai*	Prasiolaceae	Shangri-la	Whole plants	eaten fresh or added to soups	Jun-Jul		*
*Prinsepia utilis* Royle	*Qingciguo*	Rosaceae	Shangri-la, Weixi and Deqin	Seeds	used for making vegetable oil	Jul-Aug		***
*Pteridium aquilinum* (L.) Kuhn var. *latiusclum* (Desv.) Underw. ex A. Heller	*Zhila*	Pteridaceae	Shangri-la, Weixi and Deqin	Immature fronds	eaten fresh or stir-fried	May-Jul	Whole plant used to treat rheumatism or for clearing heat.	****
*Pyracantha fortuneana* (Maxim.) H. L. Li	*Sare*	Rosaceae	Shangri-la, Weixi and Deqin	Fruits	eaten fresh	Sept-Oct		*
*Pyrus betulifolia* Bunge	*Reli*	Rosaceae	Shangri-la, Weixi and Deqin	Fruits	eaten fresh	Aug-Oct		*
*Pyrus calleryana* Decne.	*Xialie*	Rosaceae	Shangri-la, Weixi and Deqin	Fruits	eaten fresh	Aug-Oct		*
*Pyrus pashia* Buch.-Ham. ex D. Don	*Suilun*	Rosaceae	Shangri-la, Weixi and Deqin	Fruits	eaten fresh	Aug-Oct		**
*Pyrus pseudopashia* T. T. Yu	*Suilun*	Rosaceae	Shangri-la, Weixi and Deqin	Fruits	eaten fresh	Aug-Sept		**
*Ramalina fastigiata* (Pers.) Ach.	*Shuhua*	Ramalinaceae	Whole plant	Whole plant	stir-fried	Jul-Sept		*
*Rheum likiangense* Sam.	*Mojue*	Polygonaceae	Shangri-la and Deqin	Young leaves	eaten fresh	Jun-Aug	Roots used to remove blood stasis.	*
*Ribes alpestre* Wall. ex Decne.	*Suanmiguoguo*	Saxifragaceae	Shangri-la, Weixi and Deqin	Fruits	eaten fresh and used to prepare local wine	Aug-Sept	Whole plants used as fence and hedge plants.	***
*Ribes moupinense* Franch.	*Hiangshen*	Saxifragaceae	Shangri-la, Weixi and Deqin	Fruits	eaten fresh and used to prepare local wine	Jul-Oct	Leaves, stems and fruits used for *weisang*. Whole plants used as fence and hedge plants.	***
*Ribes glaciale* Wall.	*Niangxu*	Saxifragaceae	Shangri-la, Weixi and Deqin	Fruits	eaten fresh	Aug-Sept	Leaves and stems used for *weisang*. Whole plants used as fence and hedge plants.	***
*Rosa omeiensis* Rolfe	*Xuwabala*	Rosaceae	Shangri-la, Weixi and Deqin	Fruits	eaten fresh	Jul-Sept	Whole plants used as fence and ornamental.	***
*Rosa praelucens* Byhouwer	*Xielermiedu*	Rosaceae	Shangri-la	Fruits	eaten fresh	Sept-Oct	Flowers used for *weisang*. Whole plant used as ornamental.	***
*Rosa soulieana* Crép.	*Xuwabala*	Rosaceae	Shangri-la, Weixi and Deqin	Fruits	eaten fresh	Aug-Sept	Whole plants used as fence and ornamental.	**
*Rubus assamensis* Focke	*Hongpai; Yongde*	Rosaceae	Shangri-la, Weixi and Deqin	Fruits	eaten fresh	Aug-Sept	Whole plants used as fence.	**
*Rubus fockeanus* Kurz	*Hongpai; Yongde*	Rosaceae	Shangri-la, Weixi and Deqin	Fruits	eaten fresh	Aug-Sept	Whole plants used as fence.	*
*Rubus niveus* Thunb.	*Hongpai; Yongde*	Rosaceae	Shangri-la, Weixi and Deqin	Fruits	eaten fresh	Aug-Sept	Whole plants used as fence.	**
*Rubus pectinellus* Maxim.	*Jiaoxumu*	Rosaceae	Shangri-la, Weixi and Deqin	Fruits	eaten fresh	Aug-Sept	Leaves and stems used for *weisang*. Whole plants used as fence.	***
*Rubus pentagonus* Wall. ex Focke	*Hongpai; Yongde*	Rosaceae	Shangri-la, Weixi and Deqin	Fruits	eaten fresh	Aug-Sept	Whole plants used as fence.	**
*Rubus polyodontus* Hand.-Mazz.	*Hongpai; Yongde*	Rosaceae	Shangri-la, Weixi and Deqin	Fruits	eaten fresh	Aug-Sept	Whole plants used as fence.	*
*Rubus rubrisetulosus* Cardot	*Hongpai; Yongde*	Rosaceae	Shangri-la, Weixi and Deqin	Fruits	eaten fresh	Aug-Sept	Whole plants used as fence.	**
*Rubus stans* Focke	*Hongpai; Yongde*	Rosaceae	Shangri-la, Weixi and Deqin	Fruits	eaten fresh	Aug-Sept	Whole plants used as fence.	**
*Sageretia thea* (Osbeck) M. C. Johnst.	*Luozi*	Rhamnaceae	Deqin	Fruits	eaten fresh	Apr-May		*
*Sambucus chinensis* Lindl.	*Debangqiongjie*	Caprifoliaceae	Shangri-la, Weixi and Deqin	Fruits	eaten fresh	Jul-Sept	Aerial parts used as fodder.	***
*Schisandra rubriflora* (Franch.) Rehder et E. H. Wilson	*Wuweizi*	Schisandraceae	Shangri-la, Weixi and Deqin	Fruits	eaten fresh and used to prepare local wine	Aug-Oct	Fruits used as antidiarrheic and for invigorating kidney. Whole plant used as ornamental.	***
*Sinopodophyllum hexandrum* (Royle) T. S. Ying	*Agabule*	Berberidaceae	Shangri-la, Weixi and Deqin	Fruits	eaten fresh	Jul-Sept	Roots, stems and leaves used to clear heat. Seeds used to cure antenatal pain and help expelling placenta. Whole plant used as ornamental.	**
*Spiranthes sinensis* (Pers.) Ames	*Xiaobaiji*	Orchidaceae	Shangri-la	Whole plant	stewed with meat and eaten as tonic	Aug-Sept	Whole plants used as fodder.	*
*Stachys kouyangensis* (Vaniot) Dunn var. *franchetiana* (H. Lév.) C. Y. Wu	*Riganlu*	Lamiaceae	Shangri-la, Weixi and Deqin	Tubers	boiled or stir-fried	Jun-Sept	Whole plants used as fodder.	*
*Taraxacum mongolicum* Hand.-Mazz.	*Yongma*	Asteraceae	Shangri-la, Weixi and Deqin	Whole plants	boiled or stir-fried	Jun-Aug	Whole plants used as fodder.	***
*Taxillus chinensis* (DC.) Danser	*Yawakeqi*	Loranthaceae	Deqin	Fruits	eaten fresh	Aug-Oct		**
*Taxillus thibetensis* (Lecomte) Danser	*Yawakeqi*	Loranthaceae	Deqin	Fruits	eaten fresh	Aug-Oct		***
*Thamnolia vermicularis* Ach.	*Xiare*	Thamnoliaceae	Shangri-la, Weixi and Deqin	Whole plant	used for making tea, wine and beverage	Aug-Oct	Used to tranquilize mind and clear heat.	*
*Thlaspi arvense* L.	*Manlancai*	Brassicaceae	Weixi and Deqin	Young stems and leaves	stir-fried or used for making pickle	May-Jun	Aerial parts used as fodder.	**
*Thlaspi arvense* L.	*Manlancai*	Brassicaceae	Weixi and Deqin	Seeds	used for making vegetable oil	Jul-Aug	Aerial parts used as fodder.	**
*Thlaspi yunnanense* Franch.	*Manlancai*	Brassicaceae	Shangri-la, Weixi and Deqin	Young stems and leaves	stir-fried or used for making pickle.	May-Jun	Aerial parts used as fodder.	**
*Thlaspi yunnanense* Franch.	*Manlancai*	Brassicaceae	Shangri-la, Weixi and Deqin	Seeds	used for making vegetable oil	Jul-Aug	Aerial parts used as fodder.	**
*Tibetia himalaica* (Baker) H. P. Tsui		Fabaceae	Deqin, Shangri-la	Roots	eaten fresh	Jun-Aug	Aerial parts used as fodder.	*
*Toona sinensis* (Juss.) Roem.		Meliaceae	Shangri-la, Weixi and Deqin	Leaf buds	eaten fresh or stir-fried	May-Jun		**
*Torreya fargesii* Franch. var. *yunnanensis* (C. Y. Cheng et L. K. Fu) N. Kang	*Shasongguo*	Taxaceae	Weixi, Shangri-la	seeds	eaten fresh or stir-fried	Sept-Oct	Leaves and stems used for *weisang*. Plants used as fuel-wood.	*
*Toxicodendron succedaneum* (L.) Kuntze	*Si*	Anacardiaceae	Weixi and Deqin	Fruits	used for making vegetable oil	Jul-Sept	Wax is extracted from fruits to use in varnish and polish.	*
*Toxicodendron vernicifluum* (Stokes) F. A. Barkley	*Si*	Anacardiaceae	Weixi and Deqin	Fruits	used for making vegetable oil	Jul-Sept	Wax is extracted from fruits for using in varnish and polish.	*
*Triosteum himalayanum* Wall.	*Sachi*	Caprifoliaceae	Shangri-la	Fruits	eaten fresh	Aug-Sept	Aerial parts used as fodder.	*
*Typhonium diversifolium* Wall. ex Schott	*Banxia*	Araceae	Shangri-la	Young leaves	used for making pickle	Jul-Aug		**
*Urtica fissa* E. Pritz.	*Yanglala*	Urticaceae	Shangri-la, Weixi and Deqin	Young stems and leaves	stir-fried	Jun-Jul		**
*Urtica mairei* H. Lév.	*Yanglala*	Urticaceae	Shangri-la, Weixi and Deqin	Young stems and leaves	stir-fried	Jun-Jul		**
*Viburnum betulifolium* Batalin	*Ruosi*	Caprifoliaceae	Shangri-la, Weixi and Deqin	Fruits	eaten fresh and used to prepare local tonic wine	Aug-Sept		*
*Viburnum kansuense* Batalin	*Ruosi*	Caprifoliaceae	Shangri-la, Weixi and Deqin	Fruits	eaten fresh and used to prepare local tonic wine	Aug-Sept		*
*Vitis betulifolia* Diels et Gilg	*Geng*	Vitaceae	Weixi and Deqin	Fruits	eaten fresh	Jul-Oct	Leaves used as fodder.	**
*Zanthoxylum bungeanum* Maxim.	*Yemu*	Rutaceae	Shangri-la, Weixi and Deqin	Young stems and leaves	eaten fresh or stir-fried	Apr-May		****
*Zanthoxylum bungeanum* Maxim.	*Yemu*	Rutaceae	Shangri-la, Weixi and Deqin	Fruits	used as spices	Jul-Sept		*****

Frequency: ***** > 75% of respondents; **** > 50% of respondents; *** > 1/4 of respondents; ** > 1/8 of respondents; * < 1/8 of respondents, but at least 2 respondents.

**Figure 2:**
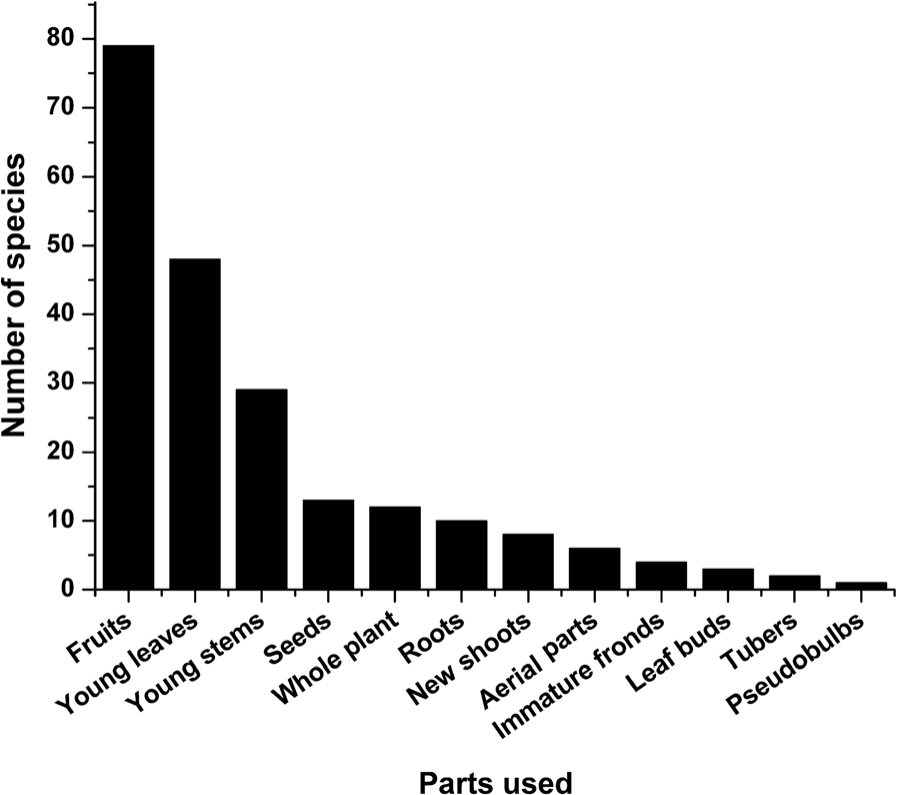
Use frequency of wild edible plant parts of species used by Tibetans in the Shangri-la region, Yunnan, China.

The most frequently used parts are fruits, young leaves and stems (Figure 2). This result is similar to other investigations, such as a study of the Shuhi people in the Hengduan Mountains (southwest China) [24], studies in Xishuangbanna, southern Yunnan (China) [26,28] and surveys among Inner Mongolian herdsmen [18]. The preference for wild collected leafy vegetables and fruits over underground plant parts seems to be common among diverse ethnic groups in China and the Himalayan area, and might be due to the ease of collecting above ground parts [24]. Collection period varies from April to August (for young leaves and stems) and July to October (for fruits and seeds). Most plant parts are collected in summer and autumn (Table 3). These plants are often dried in the sun after collection and stored (a very common preserving technique [22]) until winter. Most uses are specific to a particular plant part (such as young leaf, new shoot or ripe fruit), although in a few cases a single plant part has different uses, e.g., seeds of *Juglans regia* are eaten fresh or used to make vegetable oil. More than one plant part is used for about 7% of the species. For example, young leaves and stems of *Panax japonicus* var. *major* are used as a vegetable, while rhizomes are stewed with meat and eaten as a tonic. Leaves of *Thlaspi yunnanense* are used as a vegetable, while vegetable oil is made from the seeds. Young leaves, stems and fruits of *Berberis amoena*, *B. jamesiana*, *B. pruinosa* and *B. eisiensis* are eaten fresh. Young stems and leaves of *Zanthoxylum bungeanum* are boiled or stir-fried, and the fruits as a condiment. Fruits of *Berchemia hirtella* and *B. sinica* are eaten fresh and the young leaves to make tea. In total, vegetable (41.9%) is the most used category followed by fruit (40.8%) (Table 4). Ripe fruits are often eaten fresh, green leafy vegetative parts (e.g., young leaves and stems) are usually boiled or stir-fried, less commonly they are eaten fresh as salad or added to soups. All these plants are used as ingredients for the hot pot, since Tibetans in this region like hot pot very much.

**Table 4:** Specific edible uses of wild edible plants used by Tibetans in Shangri-la region, Yunnan, China

**Specific use**	**Number of plants**
vegetable	80
fruit	78
wine	11
vegetable oil	9
spice	8
tea	5
Total	191

These wild edible plants play an important role in providing local Tibetans with various vital nutrition elements, such as vitamins and minerals needed to maintain health and promote immunity against disease. For example, *butter rice with ginseng fruits* is a famous and traditional Tibetan dish. Ginseng fruits are the roots of *Potentilla anserina*, a perennial herb, and was reported to have low fat, high dietary fiber, all essential amino acids, various mineral elements and vitamins [41]. Other wild vegetables and fruits frequently used by local Tibetans include *Maianthemum atropurpureum*, *Allium ovalifolium*, *Aralia chinensis*, *Hippophae rhamnoides* subsp. *yunnanensis* and *Amygdalus mira*, which are all mentioned by nearly every respondent.

### Multiple uses of wild edible plants

In addition to edible use, 71.4% of the reported wild edible plants (120 species) have additional uses (Tables 3 and 5). Such species are common in rural areas and are important to local people [12,42]. They not only balance the nutritional value of starchy diets (compensating for lack of several vitamins, proteins and minerals), but may also provide pharmacologically active compounds. The multiple uses attest to the importance of these plants for subsistence and as a part of local cultural heritage [12]. Thirty-one species (18.5%) are also used as medicine, most are herbs (19 species) or trees (6 species). These medicinal plants are used to treat gastropathy, cough, fever, rheumatism, dysentery, fractures, dyspepsia, hemoptysis, and asthma. For a few species, the same part is not only used as food, but is also used for medicinal purposes. For example, the roots of *Anemone rivularis* are stewed with meat and eaten as tonic by local people, and the decoction of them are used to treat bronchitis.

**Table 5:** Types of multiple uses for edible wild plants utilized by Tibetans in Shangri-la region, Yunnan, China

**Kind of usage**	**Number of species**	**Percentage**
Edible	168	100.0
Fodder	52	31.0
Medicinal	31	18.5
Fence	22	13.1
Ornamental	11	6.5
*Weisang*^a^	10	6.0
Fuel-wood	9	5.4
Construction	4	2.4

^a^The religious rite of burning offerings for smoke, which plays an important role in local Tibetan’s daily life.

WEPs can provide resources for future exploitation of new health foods. As living standards improve, there is a globally increased demand for healthy and safe food [21]. Compared to conventional, cultivated vegetables, wild food plants require less care, are not affected by pesticide pollution, and are a rich source of micronutrients.

However destructive harvesting is a significant concern and in the present study this was documented to occur in at least 21 species used for medicine, the underground parts (root, tuber and corm) of fourteen species and the whole plant of seven species. This manner of harvest may have a serious consequence from both the survival of plants and from an ecological point of view [43]. The conservation and sustainable utilization of species with multiple uses should be taken into consideration.

Fifty-two species (31%) were used as fodder. For example *Potentilla coriandrifolia* var. *dumosa* is regarded as high-quality forage at high altitude (3500–4300 m). Further study of its nutrient composition can be done in order to understand the rationale for its usage and development potential.

Ten species have cultural significance in a religious rite named *weisang*, during which specific plants are burned for smoke. These are *Adenophora khasiana*, *Aralia chinensis*, *Cerasus conadenia*, *Pinus armandii*, *Pistacia weinmanniifolia*, *Ribes moupinense*, *Ribes glaciale*, *Rosa praelucens*, *Rubus pectinellus* and *Torreya fargesii* var. *yunnanensis*. This rite plays an important role in Tibetans’ daily life, and it is said that the fragrance in the smoke can not only make the mountain god pleased, but also wash dirty things away from people. Tibetans pray for good harvest, good fortune, happiness and prosperity in this manner.

### “*Most preferred*” species and their commercial potential

Besides food value, the recorded species provide the possibility to supplement household income of rural people with limited cash income opportunities [44]. In our survey, the most preferred plants (mentioned by more than 50% of respondents) include *Maianthemum*, *Allium*, *Aralia*, *Arundinaria faberi*, *Fargesia melanostachys*, *Pteridium aquilinum* var. *latiusclum*, *Matteuccia struthiopteris*, *Zanthoxylum bungeanum*, *Ligusticum daucoides*, *Hippophae rhamnoides* subsp. *yunnanensis* and *Pistacia weinmanniifolia*. All these plants are collected from remote mountains by local people and traded in local markets, which provides the possibility to increase the income of rural people with low cash income.

*Maianthemum* species (*zhuyecai* or “bamboo-leaved vegetable”) are the most frequently mentioned wild vegetable. In Diqing Prefecture, the leaves of six species are eaten (*M. atropurpureum*, *M. forrestii*, *M. henryi*, *M. oleraceum*, *M. purpureum* and *M. tatsienense*). They are added to soups, stir-fried with bacon or eaten raw as salad. Several studies have focused on the nutritional analysis of *zhuyecai* and found they contained higher amount of protein, essential amino acids, vitamin C and mineral elements compared with some common vegetables [45-47]. Although local people do not use them as medicine, *Maianthemum* species were reported for medicinal use since ancient times. For instance, *M. japonica* and *M. henryi* are employed to treat kidney diseases, activate blood circulation and alleviate pain [44,48]. *M. atropurpurea* contains a variety of steroidal saponins and nucleosides which may possess anti-tumor activities [49-51]. Three new steroidal saponins having cytotoxic properties against human cancer cells were isolated from *M. japonica*[52]. Z*huyecai* also has commercial value. In the market the price varied from 12 CNY (Chinese yuan) to 40 CNY (ca. 1 USD = 6.5 CNY) per kilogram (fresh weight) from April to June, an important source of cash income. And in restaurants, one dish (prepared from about 500 g) costs 18–38 CNY during another season.

Another renowned edible plant, *shutoucai,* includes two species, *Aralia caesia* and *A. chinensis*. Leaf buds and young leaves are edible and are collected from April to May. Researchers have reported that the tender shoots of *A. chinensis* contain many oleanolic acids and seven essential amino acids [53]. One local company intends to exploit this wild vegetable commercially. *Hippophae rhamnoides* subsp. *yunnanensis,* endemic to the Qinghai-Tibet Plateau, has both food and medicinal values. Its fruits are eaten fresh or used to make beverage and wine, and also used to treat cough and invigorate the circulation of blood.

In the present study, we found that taste is the first criterion for all types of food plants, in agreement with other surveys [54]. However, taste itself is not strong enough to construct a reliable priority list for future conservation, domestication and exploitation. Further detailed nutrition analysis and phytochemical investigation should be undertaken to comprehensively evaluate food and medicinal value of these “*most preferred*” plants, which could provide scientific and important information.

It is generally believed that local people are more likely to support and participate in conservation initiatives if they can receive direct benefits from such efforts [55]. If managed sustainably, these plants could be a good means of income generation for rural communities. Market surveys, value chain analyses and the risk of overexploitation should be assessed thoroughly [13,56]. *Maianthemum* populations (*zhuyecai*) are becoming rare in Shangri-la County although there were rich resources 20 years ago. Uprooting and harvesting the entire plant during collection were observed and identified as causes of decline for *Sinopodophyllum hexandrum*, *Aristolochia delavayi*, *Megacarpaea delavayi* and *Codonopsis pilosula* var. *handeliana*. Because few people in this area are aware of sustainable harvesting, the conservation and proper utilization of these species should be taught.

### Crop wild relatives for genetic improvement and crop production

Crop wild relatives (CWRs) are species that are closely related to crops including crop progenitors. These wild relatives of domesticated crops may provide genes having higher resistance to adverse circumstance that could prove particularly important in response to global climate change, which will undoubtedly alter the environmental conditions under which our crops grow and dramatically impact agriculture [4,57,58]. CWRs are also of great importance to maintain the productivity and stability of traditional agro-ecosystems [59,60]. Conservation of these species ensures that diverse genetic resources are preserved and could be used in the improvement of crops as a contribution to 21^st^ century food security [4,7,8]. The main options for CWRs conservation are *ex situ* in gene banks and *in situ* in the natural or farmed environment [59,61,62]. It is widely recognized that *in situ* is necessary to conserve the full range of genetic diversity inherent in and between plant populations, with *ex situ* techniques as a backup [58]. Taxon inventory is the starting point for *in situ* conservation which provides the baseline data critical for biodiversity assessment and monitoring [63]. Some of the wild relatives of fruit, vegetable and spice crops documented in this study are species of *Actinidia*, *Allium, Amaranthus, Amygdalus*, *Arctium, Armeniaca*, *Capsella, Cerasus, Crataegus, Dioscorea, Diospyros, Eriobotrya, Foeniculum, Fragaria, Hippophae, Juglans, Malus, Mentha, Pyrus*, *Toona*, *Vitis* and *Zanthoxylum*. Take *Amygdalus mira* as an example. Due to its advantageous traits, such as high adaptability and longevity, resistance to disease and tolerance to drought and cold, it could be a genetic resource for peach improvement. Another case is *Pyrus betulifolia*, which is usually used as stock to graft various pear cultivars. It is drought resistant, cold tolerant and long living, making it a good candidate for providing useful genes to improve the quality of pears. Young leaves of *Allium ovalifolium* could be eaten as vegetables, and leaves are relatively larger than those of other Chinese chives. Thus, it might be used as a source for breeding new variety of chives. Two other species, *Rosa omeiensis* and *R. praelucens* have edible and ornamental uses and exhibit high cold tolerance. They may provide beneficial genes for future study and exploitation in developing new crops.

### Issues of conservation

Wild edible plant species are threatened by various natural causes and human activities [4,34]. Extreme weather caused by global climate change, such as heavy snow and severe droughts, has resulted in the decrease and even loss of many wild food plant populations. Various human activities such as land use change, habitat destruction, over-harvesting and over-grazing, are major threats. In recent years, with the construction of roads, airports, reservoirs and other infrastructure, wild habitats for edible plants were severely impacted. Unsustainable harvesting of food plant species with good market price also contributes to a decrease of these plants.

Threats are not only limited to wild food plants themselves, the traditional knowledge associated with WEPs is also endangered. Therefore, systematic documentation of indigenous knowledge and biological resources is of great significance [55,64]. Along with economic development and increasing income, only a few people want to collect wild edible plants. The younger generation is becoming less interested in them, thus causing the loss of traditional knowledge. In Shangri-la County tourism is booming and local people eagerly want to serve as guides or drivers in tourist areas to pursue more money. With the convenience of transportation, residents can buy much more vegetables from the markets than ever before and do not need to collect wild species. However, in more remote rural communities where transportation is still inconvenient and people seldom go to the market, indigenous knowledge about WEPs is relatively intact. In Deqin County much land has been converted to grape cultivation to develop a wine industry and agricultural chemicals are used frequently, causing the decrease of various wild edible species, and even cultivation of the very important species, hull-less barley, *Hordeum vulgare*, the staple food of Tibetan communities [65,66] is threatened. During our survey we found that most people are reluctant to cultivate hull-less barley now because planting grapes can bring more cash income.

## Conclusion

This paper is the first ethnobotanical study of wild food plants used by local Tibetans in Diqing Tibetan Autonomous Prefecture. As plant resources in this area are rather plentiful, and under the influence of other ethnic groups, local Tibetans not only cultivate various crops, but also collect wild edible plants as food. Our survey showed the diversity of WEPs and related indigenous knowledge in this area.

Different parts of plants are used by local people, and the most frequently used parts were fruits, young leaves and stems. These plants have different specific food uses, with leafy vegetable uses being most frequent, followed by fruit uses. WEPs provide food and nutrients to local communities, such as essential amino acids, various vitamins and minerals which are needed to keep healthy and enhance immunity against diseases and infections.

If properly harvested, WEPs could be the source of cash income for local people with low cash income because they are enjoyed by local people very much and often traded in markets. Furthermore, with the increased demand for green, healthy and safe food in modern society, wild food resources have attracted global interest because they are pollution-free and contain numerous important micronutrients and pharmacologically active substances. In order to properly utilize the wild food resources, we have some suggestions: 1) properly exploit and improve conservation and management of wild food plants; 2) focus on scientific research on wild food resources; 3) protect the natural environment and habitat for wild food plants.

In addition to food value, more than 50% of recorded plants have medicinal, ornamental, and cultural and other uses that are important in local Tibetan culture. Furthermore, some are crop wild relatives and could provide useful genes for crop improvement, which may have significant consequence on global food security. However, along with the development of economy, these multi-valued resources are threatened by human activities and natural causes, and associated traditional knowledge is eroding rapidly. Therefore, sustainable management of these resources as well as conserving biodiversity is of the utmost importance.

In a word, our ethnobotanical surveys provide data and information basis for conservation and sustainable utilization of local wild edible plants, and also contribute to preserve cultural and genetic diversity in Diqing Tibetan Autonomous Prefecture.

## Competing interests

The authors declare that they have no competing interests.

## Authors’ contributions

CLL designed the study. YJ, BL and JXZ performed the field survey. YJ drafted the manuscript, BL revised the manuscript. CLL revised and finalized the manuscript. All authors read and approved the final manuscript.
